# Fully connected-convolutional (FC-CNN) neural network based on hyperspectral images for rapid identification of *P. ginseng* growth years

**DOI:** 10.1038/s41598-024-57904-3

**Published:** 2024-03-26

**Authors:** Xingfeng Chen, Hejuan Du, Yun Liu, Tingting Shi, Jiaguo Li, Jun Liu, Limin Zhao, Shu Liu

**Affiliations:** 1 Aerospace Information Research Institute, Chinese Academy of Sciences, Beijing, 100094 China; 2 State Key Laboratory Breeding Base of Dao-di Herbs, National Resource Center for Chinese Materia Medica, Chinese Academy of Chinese Medical Sciences, Beijing, 100700 China; 3 The School of Information Engineering, Xizang Minzu University, Xianyang, 712089 China; 4 The 54th Research Institute of China Electronics Technology Group Corporation, Shijiazhuang, 050000 China; 5 Jilin Provincial Key Laboratory of Chinese Medicine Chemistry, Changchun Institute of Applied Chemistry, Chinese Academy of Sciences, Changchun, 130022 China

**Keywords:** FC-CNN, *P. ginseng*, Hyperspectral images, Spectral importance, Identification, Optical spectroscopy, Optical imaging, Drug regulation

## Abstract

*P. ginseng* is a precious traditional Chinese functional food, which is used for both medicinal and food purposes, and has various effects such as immunomodulation, anti-tumor and anti-oxidation. The growth year of *P. ginseng* has an important impact on its medicinal and economic values. Fast and nondestructive identification of the growth year of *P. ginseng* is crucial for its quality evaluation. In this paper, we propose a FC-CNN network that incorporates spectral and spatial features of hyperspectral images to characterize *P. ginseng* from different growth years. The importance ranking of the spectra was obtained using the random forest method for optimal band selection. Based on the hyperspectral reflectance data of *P. ginseng* after radiometric calibration and the images of the best five VNIR bands and five SWIR bands selected, the year-by-year identification of *P. ginseng* age and its identification experiments for food and medicinal purposes were conducted, and the FC-CNN network and its FCNN and CNN branch networks were tested and compared in terms of their effectiveness in the identification of *P. ginseng* growth years. It has been experimentally verified that the best year-by-year recognition was achieved by utilizing images from five visible and near-infrared important bands and all spectral curves, and the recognition accuracy of food and medicinal use reached 100%. The FC-CNN network is significantly better than its branching model in the effect of edible and medicinal identification. The results show that for *P. ginseng* growth year identification, VNIR images have much more useful information than SWIR images. Meanwhile, the FC-CNN network utilizing the spectral and spatial features of hyperspectral images is an effective method for the identification of *P. ginseng* growth year.

## Introduction

*Panax ginseng* (family Araliaceae) is an herb that is very popular all over the world^1–4^. This species is a good source of several bioactive components (e.g., phenols, proteins, alkaloids, vitamins, ginsenosides, amino acids, etc.)^5,6^. Ginsenosides are the main bioactive compounds in P. *P. ginseng*, which have capabilities to inhibit the ROS (reactive oxygen species), production of nitric oxide and also maintain blood circulation^7–12^. More than 100 ginsenosides have been reported from different plant parts of the species (i.e., rhizomes, roots, stems, leaves, fruits, and flowers) showing a variety of therapeutic effects including anti-inflammatory, anti-allergic, anti-cancer, and anti-diabetic^13–23^. Previous studies have reported the pharmacological and physiological significance of the species, which has been traditionally consumed to enhance physical fitness, improve endurance, provide energy, etc. The production of wild *P. ginseng* is low and the current large supply of *P. ginseng* is dominated by garden cultivated *P. ginseng*^6^. In China, cultivated *P. ginseng* that is 5 years old and less than 5 years old is used as food, and cultivated *P. ginseng* that is more than 5 years old is used for medicinal purposes^24^. The accumulation of active ingredients in *P. ginseng* of different growth years is different, and the medicinal and economic values vary greatly, so it is very important to identify the growth years of *P. ginseng* by reliable technical methods.

The traditional manual identification of the growth year of *P. ginseng* is by observing the appearance characteristics such as roots, fibrous roots, and rhizomes^25,26^, which is time-consuming, laborious, and highly subjective. Therefore, it is more objective to utilize scientific instrumental methods to identify the growth year of *P. ginseng*. For example, microscopic identification can identify the growth age of *P. ginseng* by the content of calcium oxalate clusters in the rhizome^27^, and mass spectrometry^28^ and liquid chromatography^26,29^ can identify the growth age of *P. ginseng* by the active ingredients such as saponins. However, these methods are time-consuming and destructive, requiring complex pre-treatment and skilled operators. In addition, these methods are only applicable to laboratory conditions and are inefficient, making them difficult to carry out on a large scale.

Hyperspectral imaging (HSI), as a non-chemical and non-destructive technique that acquires one-dimensional spectral information and two-dimensional image information, offers significant advantages in the comprehensive analysis of samples. In the past few years, HSI has received increasing attention for quality assessment and species classification in the fields of agriculture^30–32^, food^33^ and traditional Chinese functional foods^34,35^. In a study on the classification of traditional Chinese functional foods using hyperspectral imaging, Ru et al.^36^ proposed a data fusion method in the visible-near-infrared and short-wave infrared spectral ranges and obtained a 97.3% accuracy for the classification of the geographic origin of Atractylodes macrocephala. Xia et al.^37^ investigated the effect of different wavelength selection methods on the identification of different sources of Japanese maitake, with an optimal accuracy of 99.1%. HIS provides three-dimensional information including spectrum, space and radiation. However, most of the current research methods only utilize its spectral features and do not make full use of the spatial information of its images; in addition, the radiometric information is not sufficiently utilized because of the imperfect methods of relative and absolute radiometric correction of the radiometric information^38^.

Effective analysis of the huge amount of data acquired from hyperspectral imaging is a great challenge that hinders its application. Currently, machine learning methods and deep learning methods have been developed and are considered ideal for processing and analyzing hyperspectral images. Given the unique self-learning capability and excellent performance of neural network methods, they have been widely welcomed by researchers and have been applied to the processing of spectral and hyperspectral images^39,40^ and remote sensing data.

The above studies are useful for identifying the year of growth of *P. ginseng*. However, these studies could not learn both spectral and spatial features of HSI in one model. To deal with this multidimensional and multistep classification problem, we propose a fused Fully Connected Neural Network (FCNN) and Convolutional neural network (CNN) model to synthesize HSI spectral and spatial features, named FC-CNN network, aiming at exploring the feasibility of utilizing hyperspectral images of two different spectral ranges, neural networks, and data fusion to discriminate the year of *P. ginseng* growth.

## Materials

### Sample collection and preparation

The collection of ginseng plant material is carried out according to institutional, national, and international guidelines. All methodologies are carried out in accordance with relevant institutional, national and international guidelines. Ginseng samples are professionally certified and provided by Professor Zhang Xiaobo of the China Academy, of Chinese Medical Sciences.

A total of 84 *P. ginseng* samples were collected from Jilin, Liaoning, Heilongjiang provinces, covering all main production regions in China, and their growth years ranged from 1 to 7 years. The sample numbers corresponding to different growth years of 1 to 7 years are 17, 11, 12, 12, 8, 12, 12.

### Hyperspectral image acquisition

Hyperspectral images were acquired by a hyperspectral imaging system, which contains of HySpex VNIR-1800 and SWIR-384 hyperspectral cameras (Fig. S1). The HySpex VNIR-1800 and SWIR-384 hyperspectral cameras are push broom instruments that collect spectral data in the 400–1000 nm range and 930–2500 nm range, respectively.

A platform integrated the two hyperspectral cameras. For the platform, two 150 W illumination system were used. This source features a DC regulation scheme providing stable light output intensity; eliminating output fluctuations caused by alternating current or changes in line voltage. A conveyer belt driven by a stepper motor was used for *P. ginseng* samples motion. A darkroom was used for imaging of *P. ginseng* samples. The scanned background is black. A whiteboard with Lambertian reflectance was scanned together with *P. ginseng* samples for absolute and relative radiometric correction.

A raw hyperspectral image acquired by the hyperspectral imaging system includes 288 bands for VNIR image and 108 bands for SWIR image, respectively. In total, the data size of one *P. ginseng* sample is about 2 gigabytes. Figure S2 shows the true color image in the VNIR band and the false color composite image in the SWIR band. The image in Fig. S2 clearly shows the shape and texture features of *P. ginseng* sample, as well as the flare noise at the edge of the whiteboard. Figures S1 and S2 are included in the supplementary materials.

## Methodology

**Figure 1:**
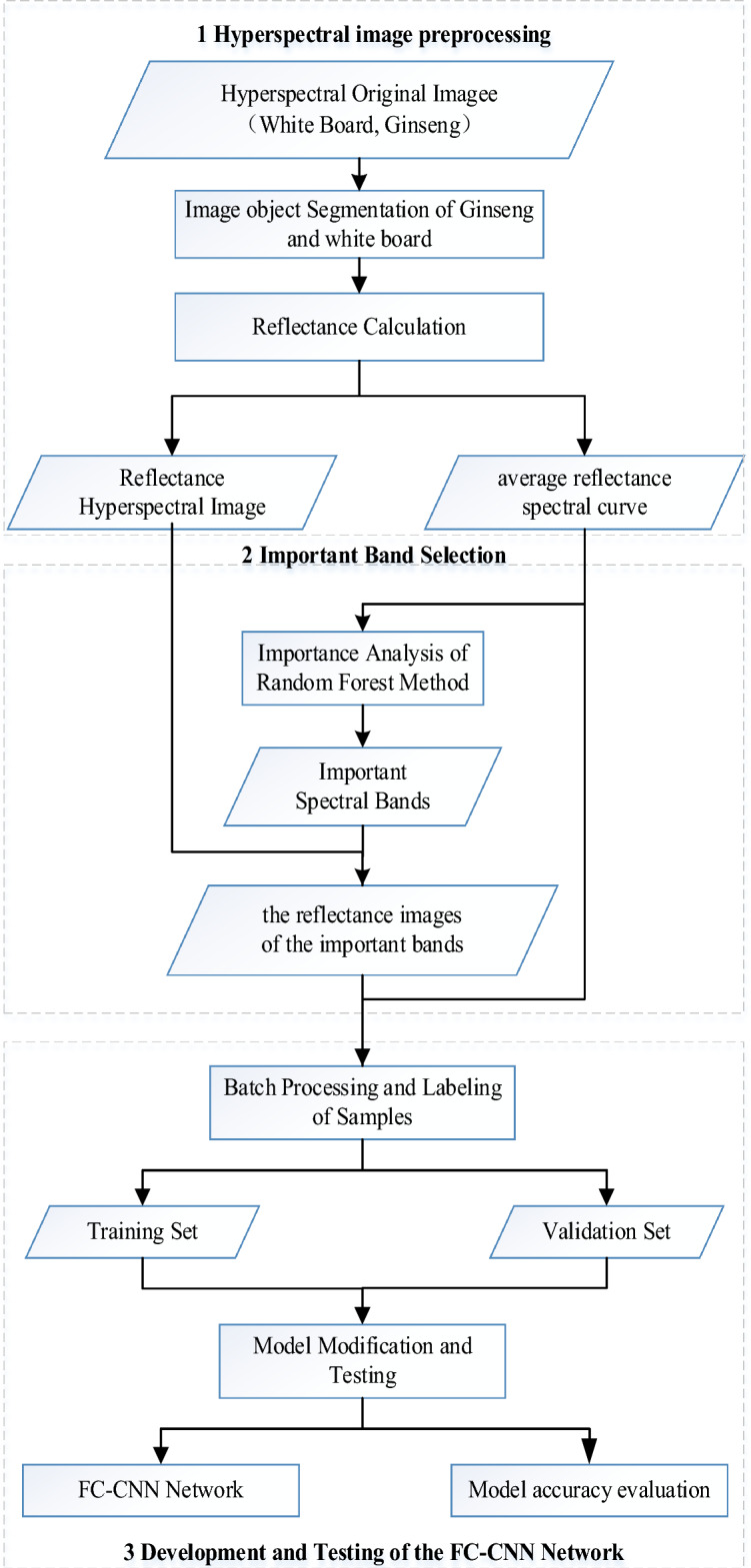
A system of methods for identifying the year of *P. ginseng* growth.

This section describes the methodological system for *P. ginseng* growth year identification using graph-spectral information from hyperspectral images, as shown in Fig. 1. The method system mainly consists of three steps: (1) obtaining *P. ginseng* spectral information: pre-processing the original hyperspectral data to obtain *P. ginseng* hyperspectral reflectance images and reflectance spectral curves; (2) obtaining *P. ginseng* graph information: extracting several bands with the greatest importance to form a new *P. ginseng* image through band optimization; (3) constructing a model for *P. ginseng* growth year identification: using the graph-spectrum information and fusing two deep learning algorithms, FCNN and CNN, to construct a *P. ginseng* growth year identification model and verify the accuracy.

### P. ginseng spectrum information

The acquisition of *P. ginseng* spectral information includes three data processing processes: (1) image segmentation; (2) radiometric calibration; and (3) reflectance spectrum calculation.

In the first step: *P. ginseng* samples and white plates are extracted from the original hyperspectral images. In the second step: the original hyperspectral images of *P. ginseng* samples are converted from digital number (DN) to surface reflectance. In the third step: the average reflectance profile of each band is calculated for each *P. ginseng* sample.

#### Image segmentation

The segmentation of *P. ginseng* samples, whiteboard and background is crucial for accurately extracting spectral information. Firstly, each *P. ginseng* sample is defined as a *P. ginseng* region of interest (GROI), and the corresponding whiteboard is defined as a whiteboard region of interest (WROI). Then, a mask was built by conducting image binarization on the gray-scale image at 622 nm and 1597 nm for VNIR images and SWIR images, respectively. The mask was used to extract GROI and WROI, as shown in Fig. S3. Pixel-wise spectra within each ROI were extracted. The head-to-tail spectra with high random noise levels were first removed. Figure S3 in supplementary materials.

#### Radiometric calibration

The purpose of radiometric calibration is to eliminate the interference of the sensor itself and convert the DN value recorded in the original image into the true reflectance data, which is an indispensable step in *P. ginseng* hyperspectral image analysis. In this study, a radiometric calibration method for *P. ginseng* hyperspectral images was defined based on the principle of hyperspectral imaging system and the characteristics of the original hyperspectral images.

Firstly, based on the Lambertian reflectance properties of the whiteboard and the fact that its reflectance is stable over the imaging time (3 days), the whiteboard can be used as a standard reference for absolute radiometric calibrations. Also, the edge pixels of the whiteboard are eliminated due to the flare in the image.

Secondly, as can be seen in Fig. S2, the pixels of each *P. ginseng* sample acquired by the hyperspectral imaging system are under different illumination and geometric conditions along the scanning direction. As can be seen in Fig. S2, due to the illumination and optical lens differences along the width direction, columns in the image have different brightness, which are bright in the center columns and dark in edge columns.

Finally, since the relative positions of GROI and WROI in the image are fixed, the reflectance of GROI is defined as:

1 $$\rho_{{\lambda \left( {i,j} \right)}} = { }\frac{{DN_{{GROI\left( {i,j} \right)}} }}{{E\left( {DN_{{WROI\left( {:,{ }j} \right)}} } \right)}}$$

where ${\rho }_{\lambda }$ represents the reflectance at wavelength $\lambda$, $i,j$ are the rows and columns of pixels in the GROI, $E({DN}_{WROI(:, j)})$ is the mean of all DNs in the column $j$ in the whiteboard, ${DN}_{(i,j)}$ is the DN located at $(i,j)$ in the GROI.

When the DNs of GROI were processed column by column using Eq. (1), the reflectance images of the *P. ginseng* samples were obtained after absolute and relative radiometric calibrations.

#### Reflectance spectral curves

The reflectance of each pixel above the GROI in the hyperspectral image of *P. ginseng* obtained by the hyperspectral imaging system differs due to the different lighting conditions at different angles of the *P. ginseng* sample surface during the imaging process. It is not rigorous to calculate the average hyperspectral reflectance profile of GROI by just selecting a certain number of sample points randomly on the surface of GROI. In this study, a method to calculate the hyperspectral reflectance curves of *P. ginseng* was proposed.

The hyperspectral reflectance images of the *P. ginseng* samples after the previous radiometric calibration process included 288 bands of VNIR images and 108 bands of SWIR images, for a total of 396 bands. A separate GROI image is generated for each band, i.e., a single-band GROI image, and the average reflectance value of the GROI on that band is found using Eq. (2). By analogy, 396 average reflectance values can be found. The 396 average reflectance values are plotted into a curve, which is the hyperspectral reflectance curve of GROI.

The average reflectance of single-band GROI images was calculated as

2 $$\rho_{\lambda I} = \frac{{\sum \rho_{{\lambda \left( {:,:} \right)}} }}{{N_{GROI} }}$$

where ${\rho }_{\lambda I}$ is the average reflectance of a single-band GROI image with wavelength $\lambda$. On the right side of Eq. (2), the numerator represents the sum of reflectance of all pixels and the denominator represents the number of pixels in the GROI image. By sequentially processing the 396 bands of the *P. ginseng* hyperspectral images, one corresponding average reflectance spectral curve can be obtained for each *P. ginseng* sample.

### P. ginseng image information

In addition to spectral information, *P. ginseng* hyperspectral images also include information such as shape and texture, which need to be obtained using images. Since the *P. ginseng* hyperspectral image contains 396 bands, if the image composed of all the bands is involved in deep learning, it will inevitably affect the computational efficiency because of the large amount of data, thus the hyperspectral image information needs to be processed for band optimization.

In this study, 288 bands of VNIR images and 108 bands of SWIR images were ranked by random forest (RF) algorithm for band importance, respectively. Based on the ranking results, the top 5 bands with the highest importance were selected and the corresponding VNIR and SWIR images were synthesized as image data for *P. ginseng* growth year identification, respectively. After band optimization of the images, the data size of each GROI is compressed from 2 GB to 8 MB.

### Model construction for P. ginseng growth year identification

#### FC-CNN model

**Figure 2:**
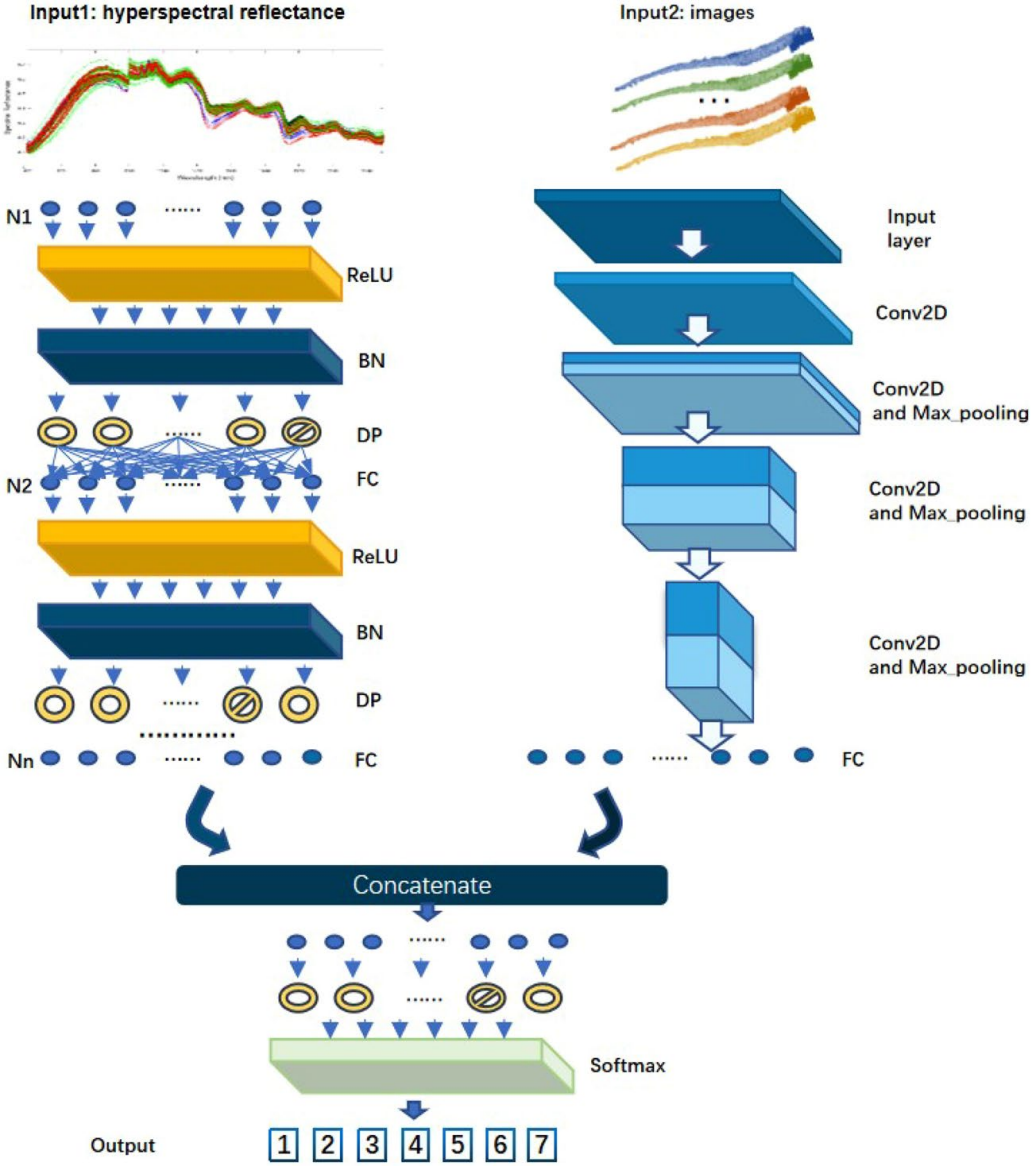
The architecture of FC-CNN network. ReLU is the activation layer. BN is a batch normalization layer. DP is the dropout layer. FC is the full connected layer. The input vector possessed N features. Nn is the neutron number of every FC layer, which could be different. Softmax is the special activation function for s classification network.

Figure 2 presents the framework of the FC-CNN model, which is comprised of two main branches: an FCNN-based spectral extractor, and a CNN-based image extractor. These two branches were finally concatenated in the fully connected layer, and the year of *P. ginseng* growth was finally output by “softmax” layer.

The hyperspectral reflectance data of *P. ginseng* samples with different growth years were input to the FCNN-based spectral extractor, as depicted in the left of Fig. 2, which extracted the spectral features of *P. ginseng* with different growth years. Note that the FCNN-based spectral extractor only exploits the spectral information. Meanwhile, the hyperspectral image data of *P. ginseng* with different growth years after band optimization were input to the CNN-based image extractor, as depicted in the right of Fig. 2, which extracted the image features of *P. ginseng* with different growth years. It is worth mentioning that not all of the five VNIR and five SWIR bands, which were preferentially selected by Section “*P. ginseng* image information”, were used in the *P. ginseng* image feature extraction, and the specific bands involved in the calculation were based on the comparison of multiple experimental results.

The innovation of our model is two-fold: (1) The spectral extractor and image extractor handled spectral information and image information, respectively, leading to an effective and interpretable prediction result. The FCNN-based spectral extractor fed with hyperspectral reflectance data of *P. ginseng* for different growth years, which could handle the spectral sensitivity of *P. ginseng* growth years. Meanwhile, the image extractor combined the predictions of the spectral extractor. (2) We selected the most important 10 bands based on band importance of 5 VNIR and 5 SWIR to combine the image data, instead of using data from all bands, which averted over-fitting and mitigated the computational challenge.

#### Model architecture

The FCNN is designed as spectral extractor which input is hyperspectral reflectance. This branch of FC-CNN model is composed of layer-group including full connected layer, activation layer, batch normalization layer and drop out layer. The architecture is shown as Table 1.

**Table 1:** The architecture of FCNN-based spectral extractor.

Layer	Layer shape (output shape)	Weights number
Input	(None, 396)	0
Dense	(None, 512)	203,264
Dropout	(None, 512)	0
Dense	(None, 1024)	525,312
Dropout	(None, 1024)	0
Dense	(None, 512)	524,800
Dropout	(None, 512)	0
Dense	(None, 128)	65,664

The CNN is designed for extracting spatial information. This branch is composed of convolution, max pooling and drop out layers. The architecture is shown in Table 2.

**Table 2:** The architecture of CNN-based image extractor.

Layer	Layer shape (output shape)	Weights number
Input	(None, 256, 1024, 6*)	0
Conv	(None, 256, 1024, 64)	3520
BatchNorm	(None, 256, 1024, 64)	256
MaxPooling	(None, 128, 512, 64)	0
Conv	(None, 128, 512, 128)	73,856
BatchNorm	(None, 128, 512, 128)	512
MaxPooling	(None, 64, 256, 128)	0
Conv	(None, 64, 256, 128)	147,584
BatchNorm	(None, 64, 256, 128)	512
MaxPooling	(None, 32, 128, 128)	0
Conv	(None, 32, 128, 64)	73,792
BatchNorm	(None, 32, 128, 64)	256
MaxPooling	(None, 16, 64, 64)	0
Conv	(None, 16, 64, 32)	18,464
BatchNorm	(None, 16, 64, 32)	128
MaxPooling	(None, 8, 32, 32)	0
Flatten	(None, 8192)	0
Dropout	(None, 8192)	0
Dense	(None, 512)	4,194,816

*The number of input images was tried in 0–6. Here only show the case that the CNN used maximum 6 images of different spectral bands.

After two branches, all features extracted from spectrum and image are combined with a concatenate layer. Then, the combined features are processed by a FCNN to the output. The architecture of the last FCNN part is shown in Table 3.

**Table 3:** The architecture of the combining FCNN to output.

Layer	Layer shape (output shape)	Weights number
Concatenate	(None, 640)	0
Dropout	(None, 640)	0
Dense	(None, 7)	4487

#### Evaluation metric

For the evaluation of the identification of the year of *P. ginseng* growth, the accuracy (the ratio of the number of correct predictions to all predictions) based on the confusion matrix is used as an evaluation metric. The formula for quantitative assessment is as follows:

3 $$Accuracy = \frac{TP + TN}{{TP + FP + FN + TN}}{ }$$

where TP is the classified accurate positive class, FP is the misclassified positive class, TN is the classified accurate negative class, and FN is the misclassified negative positive class.

#### Loss function

In this study, mean absolute error (MAE) is chosen as the loss function for multi-classification tasks. The MAE loss function compares the predicted class with the target class for each pixel, and its expression is as follows:

4 $$MAE = \frac{1}{N}\mathop \sum \limits_{i = 1}^{N} \left| {ys_{i} - y_{i} } \right|{ }$$

where $N$ is the number of samples, ${ys}_{i}$ is the $i$ th actual value, and ${y}_{i}$ is the $i$ th predictive value.

## Results

### P. ginseng hyperspectral reflectance

**Figure 3:**
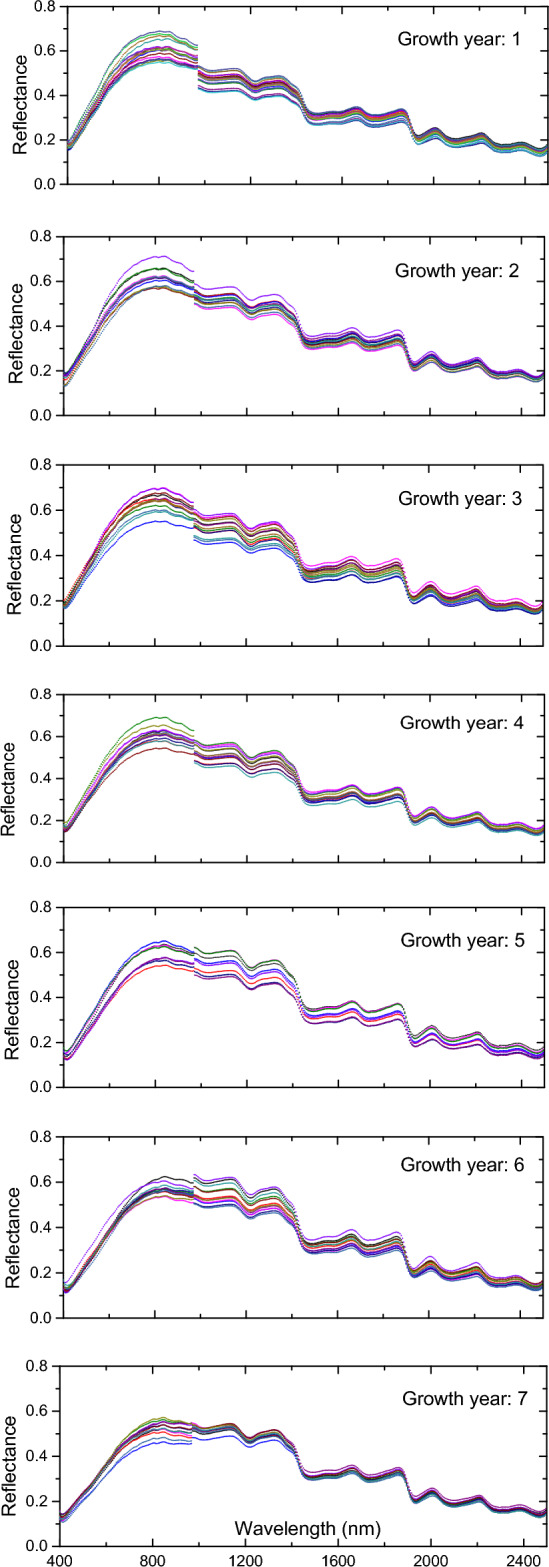
Spectral curves of *P. ginseng* samples plotted year by year.

Firstly, the reflectance of the 84 *P. ginseng* samples was obtained by radiometrically correcting the hyperspectral images of the *P. ginseng* samples according to Eq. (1). Secondly, the average reflectance of each sample in 396 bands was calculated according to Eq. (2) and plotted as a reflectance spectral curve. In order to study the spectral characteristics of *P. ginseng* in different growth years, the reflectance spectral curves of 84 *P. ginseng* samples were categorized year by year according to 1–7 years, as shown in Fig. 3.

The reflectance of *P. ginseng* samples from different years was discretely distributed over a wide range of 0.15–0.7. Therefore, there is no obvious reflectance threshold to identify the growth year of *P. ginseng*. However, the spectral curves of *P. ginseng* samples from different years showed a discontinuity near the wavelength of 1000 nm, which was caused by the different irradiation angles of the VNIR and SWIR hyperspectral cameras. By comparing the reflectance curves of *P. ginseng* from seven years, it was found that the difference between the reflectance of VNIR and SWIR at wavelength 1000 nm gradually decreased with the increase of the year of *P. ginseng* growth. The reflectance profiles of *P. ginseng* of different years varied with its molecular and chemical properties^1^.

In the reflectance curves of *P. ginseng* samples of the same year, in the range of VNIR wavelengths, the differences in reflectance between different samples increased with the longer wavelengths, and the reflectance of all *P. ginseng* samples reached the peak at 800 nm, and the differences in reflectance between different samples were also the largest; in the range of SWIR wavelengths, the differences in reflectance between different samples were smaller with the longer wavelengths. The reflectance spectral curves of all *P. ginseng* samples of different years were input into the FCNN branch of the FC-CNN model as spectral data for the identification of the year of *P. ginseng* growth.

### Important bands selection

As can be seen from the previous section, in the wavelength range of 400–2400 nm, VNIR (400–1000 nm) has 108 spectral bands, and SWIR (1000–2400 nm) has 288 spectral bands. In order to test the spectral band importance for the identification, the training and test dataset were selected randomly for 10 times. Random forest is a machine learning method that uses multiple weak decision trees to vote to determine the output, and it can provide the importance of the input spectral band. It contains many decision trees representing a distinct instance of the classification of data inputs into the random forest model^41,42^.

**Figure 4:**
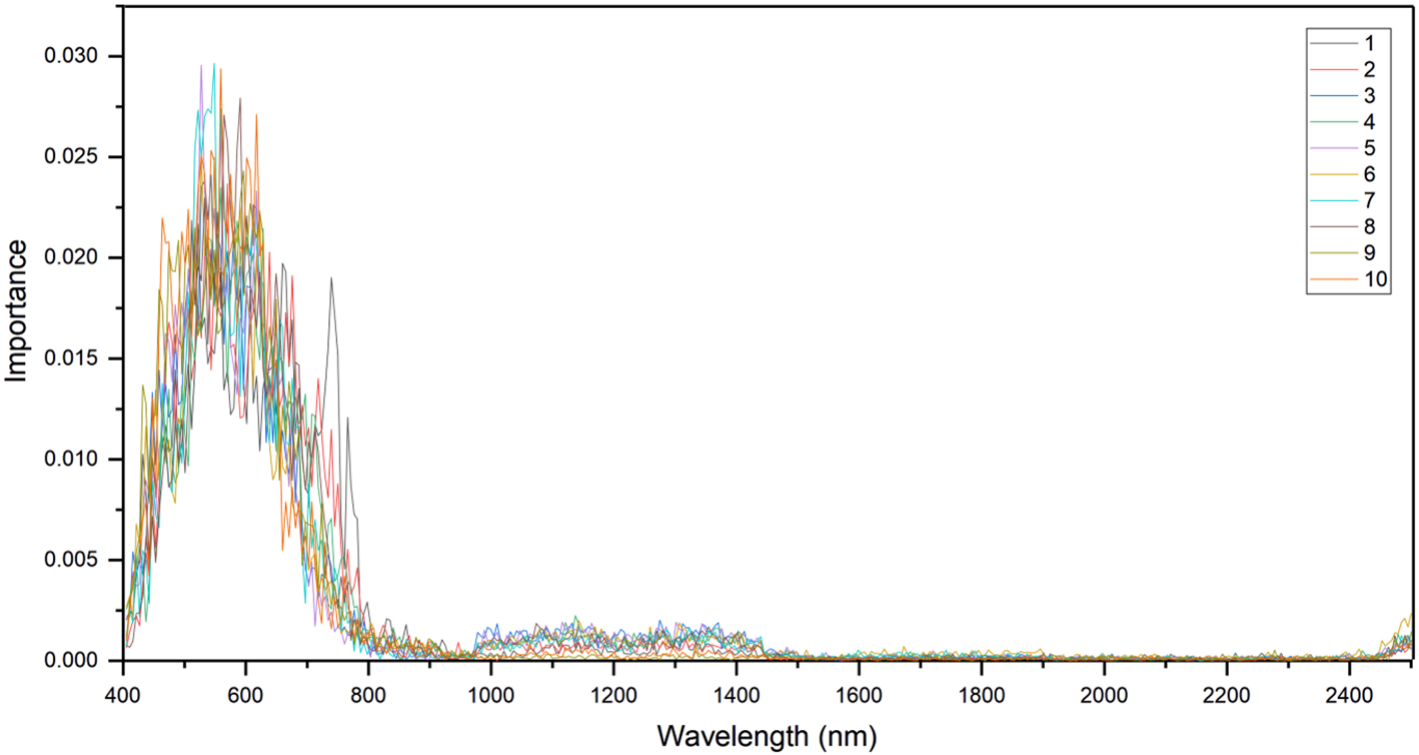
The importance of each band in 10 experiments. The color of the curve in the figure represents the number of randomized trials.

According to 10 randomized experiments, the importance of each band is obtained by ordering the importance of the bands in the RF algorithm, as shown in Fig. 4. The sum of the importance of the bands contained in each of the VNIR and SWIR accounted for 89.4% and 10.6% of the total importance of all bands, respectively. This shows that the VNIR band is more important than the SWIR band in *P. ginseng* growth year identification.

Based on the results of the importance of each band shown in Fig. 4, the five VNIR bands and five SWIR bands with the largest importance values were selected. Among these 10 bands, the images generated using different combinations of bands were input into the CNN branch of the FC-CNN model as image data for *P. ginseng* growth year recognition. Among them, the five selected VNIR bands corresponded to center wavelengths of 517 nm, 559 nm, 649 nm, 766 nm, and 898 nm, while the five selected SWIR bands corresponded to center wavelengths of 1142 nm, 1280 nm, 1412 nm, 1895 nm, and 2495 nm, respectively.

### Year-by-year identification

According to the structure of the FC-CNN model proposed in this paper, three groups of *P. ginsengs* year-by-year growth recognition experiments, named Test 1, Test 2 and Test 3, were established respectively. Each group of experiments is randomly trained and validated 10 times respectively. In Test 1, the input data were *P. ginseng* images generated from the layer stacking of five VNIR bands and 84 *P. ginseng* reflectance spectral curves; in Test 2, the input data were *P. ginseng* images generated from the layer stacking of five SWIR bands and 84 *P. ginseng* reflectance spectral curves; in Test 3, the input data were *P. ginseng* images generated by a layer stacking of three VNIR bands and two SWIR bands and 84 *P. ginseng* reflectance spectral curves. These data were fed into the FC-CNN model containing both FCNN and CNN branches, and the model outputs the classification results for the seven years of *P. ginseng*. After 10 validation tests, it can be guaranteed that the *P. ginseng* samples cover 7 different years. After 1800 iterations of each experiment, the value of the loss function gradually decreases until it converges and tends to 0, and the descending curve of the loss function gradually stabilizes.

Table 4 shows the results of the 10 tests for the three sets of trials. Table 4 visualizes that the average accuracies of the three sets of tests are, in descending order, Test 1, Test 3, and Test 2. This indicates that in the identification of the year of *P. ginseng* growth, the recognition is worst when the image information contains only SWIR bands; when the image information is a layer stacking of three VNIR bands and two SWIR bands, the average accuracy of the recognition is 3.6% higher than that of only SWIR bands; and when the image information contains only VNIR bands, the average accuracy of the recognition is another 3.5% higher than that of the layer stacking of three VNIR bands and two SWIR bands. This further verifies that the image information of VNIR bands is much more important than that of SWIR bands in *P. ginseng* growth year recognition.

**Table 4:** Validation results of FC-CNN model with 3 sets of different input data.

Rounds	Accuracy/%		
	Test 1	Test 2	Test 3
1	52.9	58.8	70.6
2	82.3	70.6	64.7
3	70.6	47.1	70.6
4	70.6	70.6	76.5
5	64.7	58.8	64.7
6	64.7	64.7	64.7
7	82.3	70.6	70.6
8	82.3	70.6	64.7
9	76.5	70.6	70.6
10	64.7	58.8	58.8
Average accuracy/%	71.2	64.1	67.7

In the 10 randomized experiments of Test 1, the highest and lowest accuracies of *P. ginseng* growth year recognition by the FC-CNN model proposed in this paper were 82.3% and 52.9%, respectively, with an average accuracy of 71.2% (Table 4).

**Figure 5:**
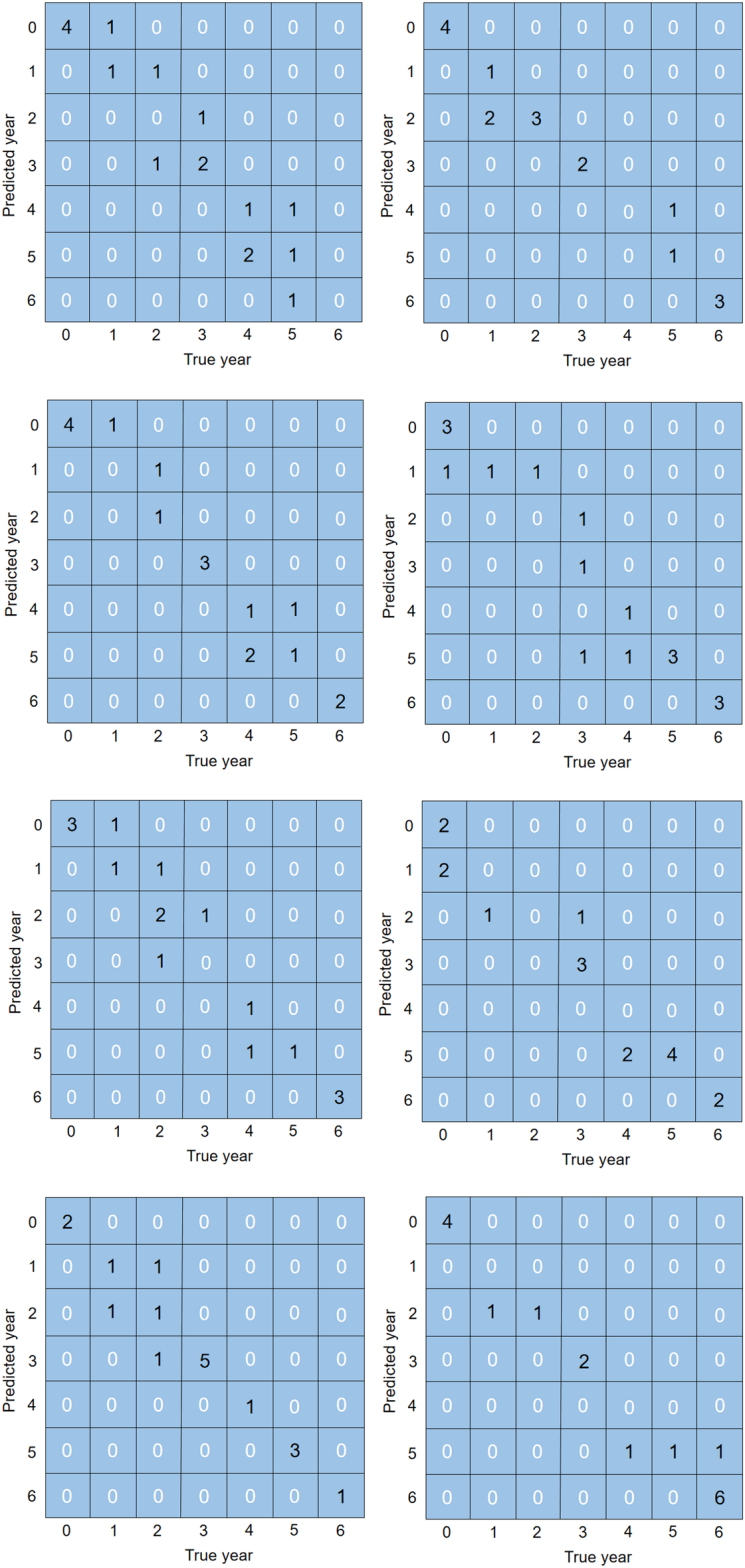

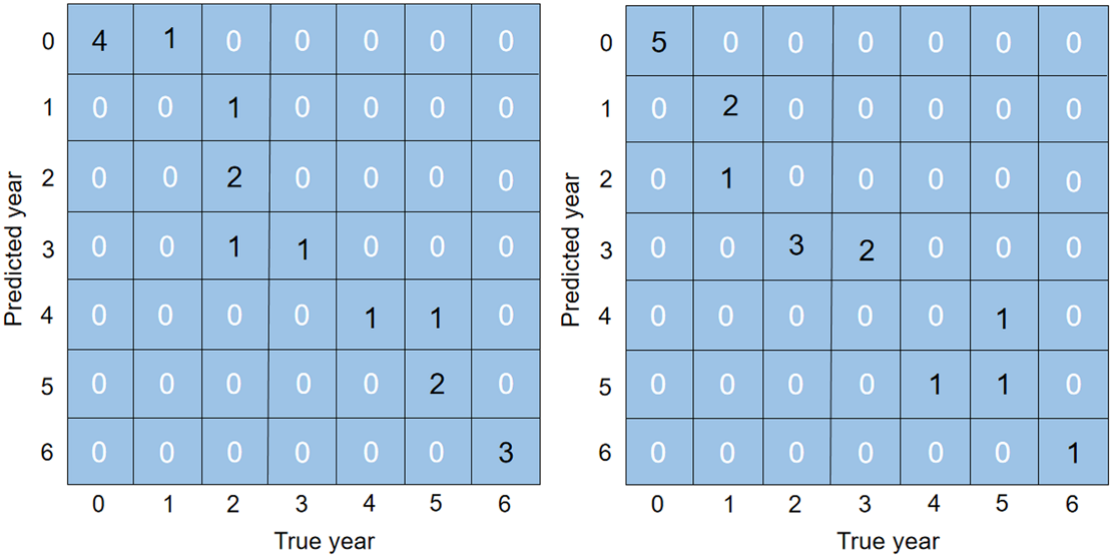
Confusion matrix results for 10 randomized trials of Test 1.

The 10 confusion matrices obtained from each of the 10 randomized trials are shown in Fig. 5. The results showed that the year prediction error rate was higher for 2-, 3- and 5-year old *P. ginseng*. Most of the misclassified *P. ginseng* growth years are susceptible to overestimation, which may be explained by the fact that the 84 samples were divided into seven categories and the sample size may be insufficient. Increasing the number of samples is necessary to improve the accuracy of *P. ginseng* growth year identification. The significance of the *P. ginseng* growth year-by-year identification experiment is not only that it can test the ability of the FC-CNN model to identify the growth year of *P. ginseng*, but more importantly, the ability of the model to carry out the identification of *P. ginseng* as a food or medicine.

### Identification of food and medicinal uses

*P. ginseng* can be categorized into food (1–5 years old) and medicinal (6–7 years old) according to the year of growth. In this paper, 7 sets of comparative experiments were designed to validate the FC-CNN model's ability to recognize *P. ginseng* for food and medicinal use, and the results are shown in Table 5.

**Table 5:** Results of food and medicinal identification of *P. ginseng* using different models.

Models	Accuracy/%
Model 1: Full Spectrums + FCNN	85.0
Model 2: Full Spectrums + Optimal 1VNIR band + FC-CNN	85.3
Model 3: Full Spectrums + Optimal 2VNIR band + FC-CNN	86.7
Model 4: Full Spectrums + Optimal 3VNIR band + FC-CNN	88.2
Model 5: Full Spectrums + Optimal 4VNIR band + FC-CNN	96.4
Model 6: Full Spectrums + Optimal 5VNIR band + FC-CNN	100.0
Model 7: Full Spectrums + Optimal 6VNIR band + FC-CNN	100.0

Before these experiments of FC-CNN models, CNN model only inputting regular RGB (622 nm, 546 nm and 443 nm spectral bands) had been tested for the RGB image is the most popular format and easiest to obtain in VNIR bands. The RGB-CNN model achieved an accuracy of 82.3%, which shew a worse performance than only HIS-FCNN model as shown in Table 5 Model 1. For a higher application accuracy, the HSI spectrums were jointly used together with images VNIR bands by FC-CNN model. Recognition of *P. ginseng* for food and medicinal use varies by numbers of optimal VNIR band. The accuracy of *P. ginseng* food and medicinal identification varies as the images with different numbers of optimal VNIR band are fed into the FC-CNN model, as shown in Table 5. It can be clearly seen that as the numbers of optimal VNIR band increases, the amount of image information that can be learned by the FC-CNN model increases, and the recognition accuracy of *P. ginseng* for food and medicinal purposes increases. Of course, as the amount of data increases, therefor does the time it takes the computer to run. When the numbers of optimal VNIR band are increased from 4 to 5 and 6, the recognition accuracy of *P. ginseng* for food and medicinal purposes can reach 100%. Generally, the Model 6 is the best choice for food and medicinal identification.

## Discussion

Compared with other commonly used identification methods for expensive Chinese Materia Medicas, FC-CNN can fulfil the requirements of high efficiency, rapidity and easy operation. At the same time, it does not damage *P. ginseng*, and professionals with non- traditional Chinese medicine background can also quickly identify the year of *P. ginseng* growth.

1. Previous methods for identifying Chinese Materia Medica in terms of species, quality, and growth year, such as microscopic identification^27^, mass spectrometry^28^, ultraviolet detection^43^, and high-performance liquid chromatography^44^, involve slicing, grinding, and liquid extraction, which can cause damage to expensive Chinese Mate-ria Medica, and greatly limit large-scale implementation of relevant identification of such Chinese Materia Medica. The FC-CNN model does not even need to touch the Chinese Materia Medica themselves, and only utilizes the spectral and spatial information of their images, which will not cause any damage to the Chinese Materia Medica being identified and will not affect their reuse.
2. Mass spectrometry^28,45^ requires a large mass spectrometer, chemical reagents, and so on, which are beyond the capabilities of non-specialized personnel. It requires professional technicians to operate in specialized laboratories. The hardware measurement system and software method of FC-CNN model support the operation by people with non- traditional Chinese medicine or chemistry background.
3. Chromatography^45,46^ requires a lot of pre-processing and consumes a lot of time. With the support of GPU-accelerated deep learning algorithms, the recognition waiting time of the FC-CNN method can reach less than 1 s, and there is still room for acceleration, with the ability of real-time recognition.

Different species contain different chemical compositions, and the characteristic spectra between them are easier to find, which are generally reflected in the absorption or reflection peaks at fixed wavelengths. On the other hand, *P. ginsengs* with different growth years belong to the same species, and the chemical composition contents may be different, but it is difficult to find the characteristic spectra reflecting the differences of *P. ginseng* years in the spectra. For the lack of characteristic spectra and differences in spectral information, it is more appropriate to choose the neural network method with strong nonlinear fitting ability to fit the relationship between spectral—image information and growth year^47^. A total of 396 absolute reflectance values were given by VNIR and SWIR in the wavelength range of 400–2500 nm, which were used to characterize the spectra of different *P. ginseng* samples; at the same time, the images of the optimal wavelength bands selected according to the Random Forest method were used to characterize the spatial features of different *P. ginseng* samples. With the support of the neural network method, the rich spectral and image information can help to ensure the high recognition accuracy of *P. ginseng* growth year.

More studies have been conducted on the identification of Chinese Materia Medica using spectral methods such as visible/near-infrared and short-wave infrared wavelength data, but fewer studies have been conducted on combining images with spectra. Due to the large amount of data, the important role of hyperspectral image information has been neglected. Therefore, the FC-CNN model proposed in this paper has more information and higher identification accuracy than methods that only utilize spectral information. In addition, the FC-CNN model maximizes the data compression by selecting the important bands, which solves the problem of large amount of network data.

Hyperspectral imaging equipment is expensive, which poses a great obstacle to the popularization and application of FC-CNN model in the *P. ginseng* plantation, trade, medicine and catering industry chain. However, according to the FC-CNN model, a combination of VNIR and SWIR can be used in the spectral dimension with imaging independence. Only multispectral bands of VNIR are needed, and full-spectral imaging is not required. This will provide the basis and model reference for simplifying the hardware manufacturing and reducing the economic cost. The accuracy and application range of the FC-CNN model is limited by the selection of training samples. Based on the available data, more samples for testing are needed to carry out method modeling and design research as well as to develop systems to support practical applications.

## Conclusion

In this paper, 84 *P. ginseng* samples with growth years ranging from 1 to 7 years were used, and after a series of data processing, the optimal VNIR and SWIR bands extracted by RF were utilized, combined with the spectral and spatial information of hyperspectral images of *P. ginseng* samples, to propose a FC-CNN model for the identification of ginseng growth years by fusing FCNN and CNN algorithms, and validated the model, and the following conclusions were obtained.

1. The useful information of VNIR image is more than SWIR image, FC-CNN model can ignore the information of SWIR image in *P. ginseng* growth year recognition, which provides an effective reference for the parameter simplification of other network models for *P. ginseng* growth year recognition.
2. In the *P. ginseng* food and medicinal identification experiments using 5 years of ginseng age as a reference, the accuracy of FC-CNN model identification can be up to 100% by only utilizing the image information of the 5 optimal VNIR bands and the reflectance spectral information of all the bands. Therefore, image information of all bands is not required in the identification of *P. ginseng* for food and medicinal use. This indicates that the method can greatly compress the amount of data involved in the computation while ensuring high identification accuracy. Even RGB image is the lowest-cost hardware solution, the RGB-CNN model can not achieve high accuracy. HSI information is important to be jointly used, and at least a 5 bands multi-spectral imaging capability is needed.
3. The FC-CNN model proposed in this paper can better recognize *P. ginseng* of different growth years, and its recognition effect for food and medicinal *P. ginseng* is superior to the FCNN branching model using only spectral information and the CNN branching model using only image information.

The FC-CNN model proposed in this paper makes full use of the spectral and image information of hyperspectral images to obtain a high recognition accuracy of *P. ginseng* growth year quickly and non-destructively. The method does not require professional technicians with background in traditional Chinese medicine and specialized laboratories. In addition, the FC-CNN model can not only realize the identification of *P. ginseng* growth year, food and medicinal purpose, but also provide a non-destructive and fast reference method for the rapid identification of other Chinese herbal species, origin, year and active ingredients. Since the neural network algorithm is limited by the sample selection, its identification mechanism is still unclear. Therefore, in the future research, the identification of the year of *P. ginseng* growth will be carried out by combining the chemical composition of *P. ginseng* and hyperspectral data, and the identification mechanism of *P. ginseng* will be analyzed.

### Supplementary Information

Supplementary Figures.

## Data Availability

The data that support the findings of this study are available from the corresponding author upon request.
