# Author Correction: Fully connected-convolutional (FC-CNN) neural network based on hyperspectral images for rapid identification of *P. ginseng* growth years

**DOI:** 10.1038/s41598-024-59638-8

**Published:** 2024-04-17

**Authors:** Xingfeng Chen, Hejuan Du, Yun Liu, Tingting Shi, Jiaguo Li, Jun Liu, Limin Zhao, Shu Liu

**Affiliations:** 1 Aerospace Information Research Institute, Chinese Academy of Sciences, Beijing, 100094 China; 2 State Key Laboratory Breeding Base of Dao-di Herbs, National Resource Center for Chinese Materia Medica, Chinese Academy of Chinese Medical Sciences, Beijing, 100700 China; 3 The School of Information Engineering, Xizang Minzu University, Xianyang, 712089 China; 4 The 54th Research Institute of China Electronics Technology Group Corporation, Shijiazhuang, 050000 China; 5 Jilin Provincial Key Laboratory of Chinese Medicine Chemistry, Changchun Institute of Applied Chemistry, Chinese Academy of Sciences, Changchun, 130022 China

Correction to: *Scientific Reports* 10.1038/s41598-024-57904-3, published online 26 March 2024

The original version of this Article omitted an affiliation for Xingfeng Chen. Their correct affiliations are listed below.

Aerospace Information Research Institute, Chinese Academy of Sciences, Beijing 100094, China.

State Key Laboratory Breeding Base of Dao-di Herbs, National Resource Center for Chinese Materia Medica, Chinese Academy of Chinese Medical Sciences, Beijing 100700, China.

The original Article has been corrected.

