# Supplementary material for: Fully connected-convolutional (FC-CNN) neural network based on hyperspectral images for rapid identification of P. ginseng growth years
Source: Sci Rep. 2024 Mar 26;14:7209. doi: 10.1038/s41598-024-57904-3 (PMC10966043; doi:10.1038/s41598-024-57904-3)
Supplement: Supplementary file 1 — Supplementary Figures. [file 41598_2024_57904_MOESM1_ESM.docx]

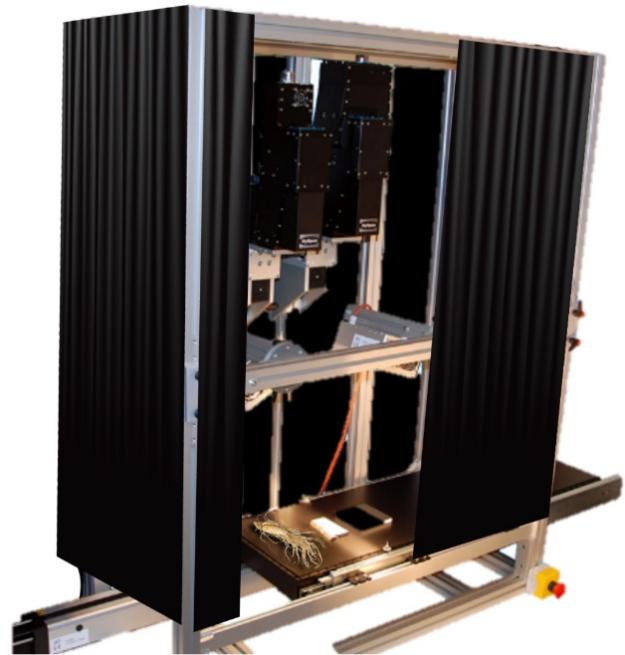


**Figure.S1:** The hyperspectral imaging system.


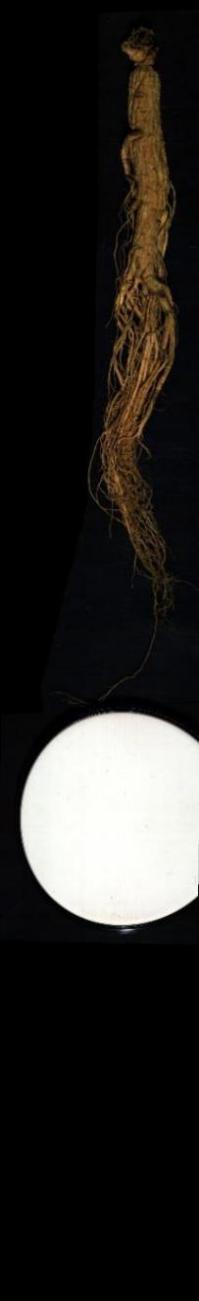

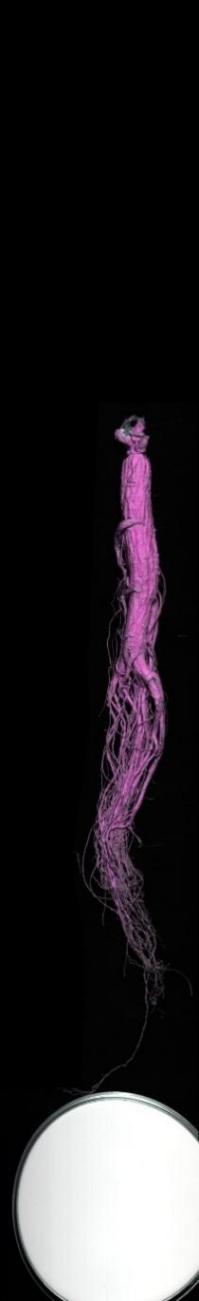


**Figure.S2:** The true color image from VNIR bands (left) and false color composite image from SWIR bands (right).


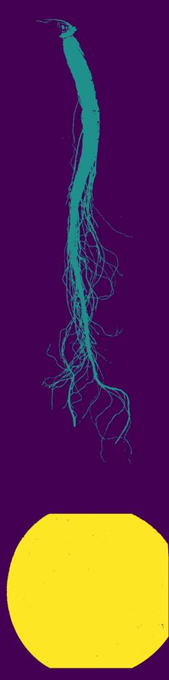


**Figure.S3:** Image segmentation results.
